# Feed handling practices, aflatoxin awareness and children's milk consumption in the Sidama region of southern Ethiopia

**DOI:** 10.1016/j.onehlt.2023.100672

**Published:** 2024-01-02

**Authors:** Anchamo Anato, Derek Headey, Kalle Hirvonen, Ashish Pokharel, Masresha Tessema, Felicia Wu, Kaleab Baye

**Affiliations:** a School of Nutrition, Food Science and Technology, Hawassa University, Ethiopia; b International Food Policy Research Institute, USA; c Department of Agricultural, Food, and Resource Economics, Michigan State University, East Lansing, MI, USA; d Food Science and Nutrition Research Directorate, Ethiopian Public Health Institute, Addis Ababa, Ethiopia; e Research Center for Inclusive Development in Africa (RIDA), Nutrition and Food Systems Division, Addis Ababa, Ethiopia; f Center for Food Science and Nutrition, Addis Ababa University, Addis Ababa, Ethiopia

**Keywords:** Aflatoxin, Dairy, Feed, Safety, Children, Nutrition, Value chain

## Abstract

Consumption of milk is linked to improved nutrient intake and reduced risk of child malnutrition in low and middle-income countries. However, these benefits are contingent on the safety and quality of the milk. Milk consumption may alleviate the widespread risk of malnutrition in rural Ethiopia, but milk-borne contaminants may also compromise child health. We aimed to: i) identify the types of dairy feeds used, their storage conditions, and potential risk of aflatoxin contamination; ii) assess stakeholders' knowledge about aflatoxin contamination along the value chain; and iii) assess parental practices on feeding milk to infants and young children.

This qualitative study was conducted in the Sidama region, southern Ethiopia. In-depth interviews (*n* = 12) and key-informant interviews (*n* = 18) were conducted with actors along the dairy value chain. Focus-group discussions were conducted with farmers (9FGD/*n* = 129) and child caregivers (9FGD/*n* = 122). Study participants were selected to represent a rural-urban gradient, as well as low- and high- dairy cow holdings.

We found that while animal-feed processors and their distribution agents had relatively good knowledge about aflatoxin, farmers and retailers did not. Feed storage conditions were poor. Many respondents linked moldy feeds to animal health but not to human health. Farmers' feed choice was influenced by cost, seasonality, and herd size. Small-holding farmers had limited access to commercial feed. Children's consumption of milk was limited to skim milk, as butter was extracted and sold for income. The high cost of dairy products also led some parents to dilute skim milk with water before feeding children, compromising the nutritional value and safety of the milk.

Our findings underscore the need to address the gaps in aflatoxin and food safety knowledge, improve storage conditions, and ensure the availability of quality feed to increase the sector's productivity, but most importantly to protect consumers' health and well-being, especially infants and young children.

## Highlights

• Knowledge gaps in proper feed storage conditions and milk handling are widespread.
• Cow feed choice is driven by cost, seasonality, herd-size and market access.
• Aflatoxin contamination is likely to vary by feed type and season.
• Dilution of milk and unsafe milk handling practices increased contamination risks.

## 1 Introduction

Chronic child malnutrition reflected by low height-for-age (i.e., stunting) affects about 149 million children worldwide [1]. Children in low- and middle-income countries (LMIC) are disproportionately affected. Low-quality diets, exposure to aflatoxin, poor childcare practices, poor hygiene and sanitation practices and a home environment that is not conducive to optimal growth are among the primary causes of the high prevalence of malnutrition in LMICs [2]. The first 1000 days from conception to the child's second birthday have been identified as a window of opportunity to prevent child malnutrition and lay the foundation for a healthy and productive life [3]. Despite the recognition of the importance of this period and numerous efforts exerted to prevent child malnutrition, the proportion of children affected by malnutrition remains high [4,5].

Studies that characterized the diet quality of infants and young children in low-income countries have shown that diets during the complementary feeding period (6–23 months) remain monotonous and predominantly cereal-based [6,7]. Despite mounting evidence linking the consumption of animal-source foods (ASF) with better child growth and a reduced risk of stunting, few children in low-income settings consume them [8]. The limited supply, relatively high cost, and perishability of milk, a key ASF with several essential nutrients for growth, are some of the bottlenecks to feeding dairy products to children [9,10]. While contamination of dairy products with bacterial pathogens is a longstanding concern in public health [11], aflatoxin M1 (AFM1) contamination of milk through animal feeds contaminated with aflatoxin B1 has emerged as a significant concern in Africa's dairy sector [[12], [13], [14]].

AFM1 is a metabolite of aflatoxin B1 (AFB1), a potent hepatocarcinogen produced by *Aspergillus* fungi in certain dairy animal feed ingredients. Methods to reduce AFB1 in field and postharvest situations [15] can also reduce the presence of AFM1 in milk. While high-quality dairy consumption could play an important role in redressing Africa's malnutrition problems, contaminated milk may attenuate those benefits, or even lead to more serious consequences for child morbidity or mortality [[16], [17], [18]].

In Ethiopia, the consumption of milk and other dairy products has been rapidly increasing over the past decade, particularly in urban areas [19]. As a result, ever more dairy farmers are switching to commercial feed to improve milk yields to meet the growing urban demand [19]. However, dairy prices outpaced general inflation by 35% in 2007–2016 [20], implying that significant bottlenecks in the dairy supply remain. Moreover, dairy productivity is low, and dairy value chains remain highly informal and weakly monitored or regulated, making food safety issues a significant concern [21]. Meanwhile, many recent studies have reported relatively high concentrations of aflatoxin M1 in milk in Ethiopia [[22], [23], [24]]. These studies have linked aflatoxin occurrence in milk to contaminated animal feeds and indicated that contamination is likely to vary by feed storage conditions, feed type, and the knowledge and practice of actors along the dairy value chain [23]. For example, animal feeds such as maize and noug cake were more susceptible to aflatoxin contamination [23]. The type of feed could vary by a farmer's market access, herd size, and education and knowledge levels [11]. However, to our knowledge, no previous studies have comprehensively investigated these issues along the dairy value chain in Ethiopia or other LMICs.

The present qualitative research aimed to identify the types of feed, storage conditions, milk handling, and consumption as well as the knowledge about aflatoxin of various actors in the dairy value chain in Sidama, southern Ethiopia.

## 2 Methods

### 2.1 Study site and participants

**Fig. 1:**
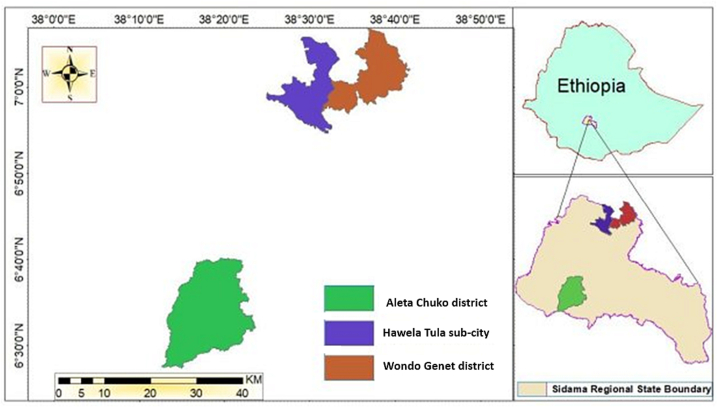
Study districts from the Sidama Administrative Region, Ethiopia.

This study was conducted in Hawassa city (Hawela Tula sub-city) and the districts of Wondo Genet and Aleta Chuko in the Sidama region, southern Ethiopia. The study sites were purposively selected to represent an urban-rural gradient with varying levels of market access (e.g. distance to markets). The study sites are shown in Fig. 1.

### 2.2 Sampling

Focus group discussions (FGDs) and in-depth/key informant interviews (IDIs/KII) participants were purposively selected with the support of development agents and health extension workers. Dairy farmers and mothers/caregivers of children were both stratified by herd-size into low-holding (owning up to two dairy cows) and high-holding (≥4 cows). Development agents work closely with farmers and can identify farmers with varying herd-size, whereas health workers identify caregivers with children less than two years of age.

### 2.3 Data collection

#### 2.3.1 Focus group discussion and interviews

A total of 18 FGDs were conducted with dairy farmers (9 FGDs/*n* = 129) and caregivers of children aged 6–23 months (9 FGDs/*n* = 122). Key informant interviews (18 KII) were conducted with feed manufacturers (*n* = 2), feed distribution agents (*n* = 4), and extension workers (n = 12). Maximum variation sampling was used, and the sample size for the FGDs was determined when saturation was reached, defined as the moment where no additional information was generated through the data collection [25].

Semi-structured interview guides contained open-ended questions that were pretested in a setting similar to the study area prior to use in the actual survey. The guides were also reviewed during the daily debriefing sessions during the data collection period. The guides included questions that assessed the type of animal feed used, aflatoxin-related knowledge of farmers, feed storage practices, and parental care practices as they related to young children's dairy consumption.

Interviews were conducted by a single interviewer, whereas FGDs were conducted by one interviewer and a note-taker. Data collection was conducted by four enumerators; Farmers were contacted two days before the FGDs and IDIs to set a date and time that would be most convenient to them. All interviews and FGDs were audio-recorded and complemented with notes taken during the discussions and interviews. The FGDs, KII, and IDIs were conducted in *Sidamigna* and Amharic languages.

#### 2.3.2 Direct observation

Direct observations of feed storage rooms, dairy farms, feed retail shops, and the household environment were conducted, allowing a direct assessment of the risk of contamination of feed.

### 2.4 Data management and analysis

The transcripts of the audio recordings were translated into Amharic (NVivo® Version 10, Burlington, M.A.) for coding and analysis. The transcribed data were openly coded by the research team members. Whenever, differences between the two coders were identified, the two coders reviewed the coding and reached consensus. The principal investigator reviewed all coding and ensured cohesion in the approach.

The data were analyzed using the content analysis method as described in [26]. Additionally, an inductive approach was used allowing for the identification of new themes during analysis [27]. Particular attention was given to how many participants shared a certain idea. Illustrative quotes were extracted and translated into English.

### 2.5 Ethical clearance

Ethical approval was granted by the Institutional Review Board (IRB Number, EPHI-IRB-481-2022) of the Ethiopian Public Health Institute, International Food Policy Research Institute (DSGD-23-0102) and Michigan State University (STUDY00007996). Informed consent was obtained from the study participants in the presence of a witness and according to the principles of the Declaration of Helsinki.

## 3 Results

### 3.1 Socio-demographic and economic characteristics of study participants

A total of 12 health extension workers and agriculture experts, 2 feed processors, 4 feed traders, 135 dairy farmers (both low- and high-holding), and 128 mothers/caregivers of children <24 months of age were included in the in-depth interviews and focus group discussions. The majority of dairy farmers were males with primary or secondary education, and were between the ages of 22 and 56 years (Table 1). Crop and livestock production were the major sources of income.

**Table 1:** Socio-demographic characteristics of the study participants in the rural, peri-urban and urban communities of Sidama, Ethiopia, 2023.

**Characteristics**	**Low holding**				**High holding**				**Key informant interviews (KIl)** (n = 18)			
	**In-depth interviews** (n = 6)		**Focus-group discussions** (n = 122)		**In-depth interviews** (n = 6)		**Focus-group discussions** (*n* = 126)					
	**Caregivers** (n = 3)	**Farmers** (*n* = 3)	**Caregivers** (*n* = 59)	**Farmers** (*n* = 62)	**Caregivers** (n = 3)	**Farmers** (n = 3)	**Caregivers** (*n* = 63)	**Farmers** (*n* = 67)	**HEWs** (n = 6)	**AEs** (n = 6)	**Processors** (*n* = 2)	**Feed retailers/** **agents** (*n* = 4)
**Sex of participants**												
Male	–	3	–	62	–	3	–	67	–	4	1	3
female	3	–	59	–	3	–	67	–	6	2	1	1
**Mean age (in years)**	33.5	42.5	32.1	44.4	31.7	39.3	29.4	43.6	27.6	41	44	34.5
	
**Mean household size**	4.3	5.3	4.7	6.0	5.6	4.6	6.2	6.6	–	–	–	–

**Educational level**												
No formal education	3	2	28	14	2	–	42	26				
Primary to secondary level education	–	2	31	48	1	2	15	29	-	-	–	–
College or university level education	–	–	–	–	–	1	6	12	6	6	2	4

**Years of experience in dairy farming**												
1–5	2		43	5	1	–	52	5	–	–	–	–
5–10	1	2	16	17	2	3	11	0.7	–	–	–	–
>10	–	1	–	40	–	–		55	–	–	–	–

HEWs: Health extension workers; DA/AEs: Development agent/agriculture experts.

### 3.2 Knowledge and awareness of aflatoxin contamination

The animal feed processors interviewed had good knowledge about aflatoxin and its health consequences. Animal feed processors reported that aflatoxin contamination increases during poor and extended storage of feed, with the knowledge that aflatoxin can be transferred to milk, posing health risks to dairy consumers. Similarly, the interviewed animal feed distribution agents knew about aflatoxin, its source, and its health consequences.

*“Aflatoxin is caused by long storage of feeds in poor conditions lacking aeration… If animals are fed moldy feed, this can harm both the animal and the humans consuming the milk…what worries me is that most of the people are not aware about the aflatoxins and its health consequences.”*

*(KII, feed processor, male, age 56 years)*

However, the animal feed retailers that were interviewed had limited knowledge about aflatoxin. Given that cattle can metabolize AFB1, they rarely manifest illness from aflatoxin contamination of feed, but as illustrated by the following quote, the feed retailer expects to hear about the sickness of the cows if his feeds were contaminated:

*“I have been in the animal feed retail business for about four years now, but I have never thought about aflatoxin or heard about it. Also, my customers have never complained that their cows got sick after eating my fodder.”*

*(KII, feed retailer, male, age 30 years)*

Similarly, almost all dairy farmers interviewed have not heard about aflatoxin, irrespective of age, number of years in the sector, and the size of their cows.

*“Many of us have been in the dairy business for many years, but we have never heard about what you are talking about [aflatoxin].”*

*(FGD, high-holding farmer, remote, male, age 39 years)*

Although the farmers did not know the term aflatoxin, they were aware of the problems associated with moldy feeds. However, moldy feed was primarily associated with poor animal health and reduced milk output, as illustrated by the following quotation:

*“If the animal consumes moldy feeds they become sick and show symptoms like diarrhea, they become sleepy and inactive, and the amount of milk the cows produce is reduced.”*

*(FGD, high-holding farmer, accessible, male, age 36 years).*

Moldy feed was considered contaminated, and according to the farmers, traits like color change, formation of clumps and fibrous material, insect infestation, and the presence of insect larvae were indicators of contamination. These indicators are not necessarily the results to aflatoxin contamination, but are those identified by farmers as indicators of feed contamination.

*“When we give contaminated feed to the animals, they refuse the feeds especially if it has an abnormal smell… we also often have animals with poor appetite, giving milk with an abnormal taste… the cows are in poor health and can eventually die.”*

*(FGD, high-holding farmer, accessible, male, age 40 years)*

### 3.3 Storage of animal feed and contamination

Despite of the association between mold development in feeds and storage conditions and consequently animal health effects, storage conditions mostly remain quite poor, particularly among feed retailers and the farmers themselves. Small retailers of animal feed keep feeds on the floor and can, at times, keep some of these commercial feeds for as long as seven months. No special aflatoxin prevention techniques were reported. Dairy farmers started stockpiling animal feeds from September to November, and there was no report of good storage practices like “first in, first out” (use the feed that has been stored for the longest first). Our direct observation of the storage conditions revealed that most farmers were storing feed in places with very limited light and air-circulation.

*“Dairy farmers store animal feed ingredients such as spent yeast, Frushka (bran/chaff) and Fagulo (Noug cake) in pool and sacks, whereas corn, barley and common bean straws are stored in iron sheet house constructed to serve as a warehouse.”*

*(IDI, male, high-holding farmer, accessible, age 30 years)*

*“There are not enough feeds we stockpile for the dry season. But the most common ones we used to store are corn straw and dried grass. We don't have any house to store these ingredients, but our community piles them on the soil between enset plantations. Most of the fodder stocks are used between October and March.”*

*(FGD, low-holding farmer, accessible, male, age 44 years)*

### 3.4 Aflatoxin prevention and mitigation measures

Although animal feed processors were aware of the negative effects of aflatoxin, they did not directly test their feeds for the presence of aflatoxin. Instead, they relied on the visual investigation of the ingredients for molds and avoided their use in making the feed mixes. The main hindrance to testing the feed was the high cost of aflatoxin tests and the inability to prevent contamination from the source, as illustrated by the following quote.

*“The majority of the food processors in Hawassa city and its vicinity do not have the capacity nor the facilities and equipment to test aflatoxin. Even if there is equipment to test, the costs of testing are very expensive. Besides, contamination begins from the raw materials and there is no way to control that… In the processing line, hygiene is usually an issue, the machines are not well cleaned, and thus contribute to the end product contamination.”*

*(KII, feed processor, female, age 29 years)*

By contrast, farmers reported sun-drying moldy feed ingredients and then feeding them to animals. A few reported picking and sorting to remove moldy ingredients. Others have reported mixing spoiled grains with seemingly good grains to dilute and minimize the potential adverse health impact. This is illustrated well by a quote from an FGD participant:

*“Because we buy animal feed at a high price, we do not throw it away if it gets moldy or contaminated; rather, we use sun-drying of the spoiled ingredient and feed it to the cows. Sometimes we use water to wash the contaminant if it is affecting a small proportion of the ingredient… this way we try to remove the mold.”*

*(FGD, high-holding farmer, accessible, age 46 years)*

### 3.5 Common animal feed ingredients and seasonality

About 24 animal feed ingredients were identified through FGDs with farmers (Table 2). These ingredients fall under i) natural pasture grazing, ii) natural grass (dried or succulent grasses), iii) improved forages (Elephant, Rhodes, Desho and Guatemala grasses), iv) concentrates (beer juice, Noug cake (*Guizotia abyssinica*), molasses, commercial feed mix, grain, barley, corn, common bean and teff straws), v) non-conventional feeds (sugar cane head cut and stem, fresh corn stalks and head cuts, enset leaves and pseudo stems, banana leave and stem, fruits and vegetables peels, common salt, as well as grass and herbs like *Shomoda, Conyza bonariensis,* and *Korchisha*, *Lalunte*.

**Table 2:**
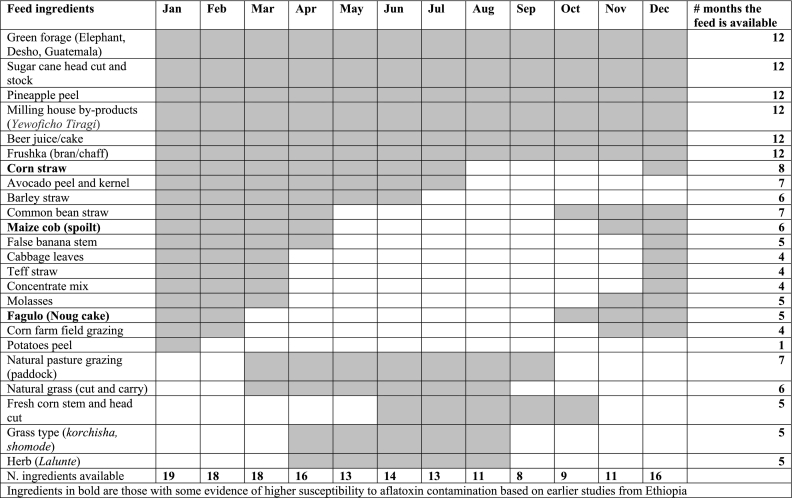
Seasonality mapping of animal feeds availability in Sidama region, Ethiopia.

Ingredients in bold are those with some evidence of higher susceptibility to aflatoxin contamination based on earlier studies from Ethiopia.

Only 6 of the 24 ingredients were reported to be available throughout the year (Table 2). Another six ingredients were available for six or more months in the year. The maximum number of feed ingredients was reported to be available between January and March, after which the type of ingredients available starts to decline to reach an all-time low of 8 to 9 feeds in September/October. The availability of concentrates overlapped with the period when most animal feed ingredients were available (January to March). In the dry season, cuts of sugar cane, banana stem, teff straw, *frushca*, water, and salt are used to prepare silage for milking cows.

### 3.6 Drivers of feed choice

This research revealed that feed cost, seasonality, accessibility, and dairy cattle herd size were the main drivers influencing the choice of feed. Study participants reported that there is a clear seasonal variation in the availability of animal feed. In the rainy season, grasses, fresh corn, and different types of weeds are available for animal feeding, and thus the probability of aflatoxin contamination is almost certainly minimal. However, in the long dry season (September to May), cows are fed from stock and free-grazed on corn and other crop fields leading to a higher probability of risk of aflatoxin contamination. Prices of purchased feed also vary predictably, rising during the dry season, but also producing substitution between different types of feeds:

*“Animal feed price and availability vary by season. For example, during the rainy season, there is enough grass and corn straw in the market, so the prices of other types of fodder like commercial feeds and Frushka are reduced. In the dry season, grasses, corn stalk and straw, and common bean straws are commonly used as animal/cow feed because their prices are cheaper than other commercial feeds. Thus, depending on the season, we select the cheaper alternatives.”*

*(IDI, high-holding famer, accessible, male, age 43 years)*

Besides grass, straw and milling by-product (*Frushka*), some participants reported feeding *Mitin* (feed-mix) to their cows, but this tended to vary by herd size, as illustrated by the following quote:

*“The price of animal feeds increases substantially during the dry season due to a mismatch between supply and demand. Having several animals, as I do, I cannot afford the skyrocketing price of the animal feeds… I opt to reduce the amount/proportion of the ingredients in the mix to be able to feed a larger number of animals and minimize my expense on commercial feeds.”*

*(FGD, high-holding farmer, accessible, male, age 33 years)*

Farmers who have large herd sizes can purchase larger amounts than farmers who have a small number of cattle. However, even for large holding farmers, the cost of feed is a major constraint that reduces their profit margin from milk sales. Due to the scarcity of animal feed, farmers focus more on the acquisition of sufficient quantity of feed rather than the quality of the feed:

 *“Animal feed is the main problem of this community. We have at least two to three cows that that need to be fed to give milk. Thus, no one is concerned about feed contamination and the resulting human and animal health problems. Getting enough feed is itself an achievement. Our main concern is to not run out of stock.”*

*(FGD, low-holding farmer, remote, male, age 42 years)*

To our surprise, we found that large holding farmers purchased feed from a wide variety of feed sellers: grass from schools, health centers, and Universities through open bids, and direct purchases from traders, agents, farmers, and retailers. In contrast, small-holding farmers mostly rely on their own production and less on commercial feed. And neither smallholders nor large holders can purchase from animal feed processors directly, as the quote below explains:

*“The animal feed processors do not sell for farmers whether the farmers have a large herd or not for two reasons. The first reason is that the farmers cannot afford large quantities of feed; the second reason is that the processors have contractual agreements with agents to distribute their products. The only option we have is to purchase from agents only.”*

*(IDI, high-holding farmer, accessible, male, age 38 years)*

Besides seasonality, cost, and herd size, physical access to feed is also a major driver. A considerable proportion of the discussants from rural settings indicated that it is hard to get commercial feed ingredients nearby except frushca, which is sold in retail shops. In contrast, dairy farmers from peri-urban areas reported that commercial feed is available, but the high price is a major constraint. Consequently, when available, commercial feed in rural areas is used as a supplement rather than as the main feed.

“*Although we would like to feed our cows with better feeds, we do not have access to commercial feeds, except for frushca that is sold in retailers' shops. If we go and buy from Hawassa Town, we will spend a lot of money, including the cost of transportation, loading, and unloading. Therefore, we are forced to limit our feed choice to fodders such as grass, corn cane, sugar cane…that we can buy from farmers in our village”.*

*(FGD, low-holding farmer, remote, male, age 45 years)*

*“We don't have all types of animal feeds at our store; however, noug cake, cotton seed cake, grains, molasses, commercial concentrate mix, and brewery juice are abundant in the local markets. However, due to their cost, we don't always purchase and feed these products as the main feed, but instead use them as a supplement to enrich common feeds”.*

*(FGD, high-holding, accessible, male, age 39 years)*

Farmers also complained about the limited access to free grazing as a main constraint leading to low milk productivity.

“*In the old days, our cows used to free graze because there were large private and government farms, as well as uncultivated land, but this is becoming rare. The only free pasture we have is corn fields that can be used for free grazing after harvest. We use these fields for about three months from Tahsas (November) to Tirr (January)”.*

*(IDI, low-holding, remote, male, age 50 years)*

### 3.7 Milk consumption among children

The main foods given to children were cereals (maize), root and tubers (kocho), pulses, and vegetables such as kale, cabbage, and tomatoes. Avocados and bananas were also fed to children. Animal source foods were rarely consumed, and milk was given only to children after extracting butter.

*“…the foods that we commonly feed our children are flatbread from maize, a small portion of injera from teff, and kocho from enset accompanied with common bean and/or kale, potatoes, and tomato stew. Meat is fed to children during holidays only.”*

(*FGD, from high-holding, remote, mother, age 31 years)*

*“Milk is primarily produced to be sold in the market either in raw or in the form of butter…if given to children, it will be after extracting the butter…the money earned from this can buy other household necessities such as salt, food, kerosene and oil…”.*

*(FGD, from low-holding, accessible, mother, age 36 years)*

The participants also mentioned that the prices of dairy products are so high that they not only sell butter but also skim milk. This is incentivizing households to dilute skim milk with water before feeding children.

“*The money earned from selling a small pack of butter and a liter of skim milk is sufficient to purchase oil, one kilogram of maize flour, and salt for the household.”*

*(IDI, from low-holding, remote, mother, age 34 years).*

*“We add water to skim milk before it is separated from butter. This is quite common in our community. It has two purposes: one is to increase the volume of the milk so that it can cover all members of the family; the other is to facilitate the extraction of butter.”*

*(FGD, from low-holding, accessible, mother, 33 years).*

*“Water is not added to milk only to increase volume and extract butter but also for income. It is common to add 1L of water to 2L of milk. In the local market, 1L of skim milk is 100 birr. Thus, I can sell 2-L milk and provide 1 L for my children. The sale of two liters of milk can cover at least two days of family food and other household necessities.”*

(*IDI, from high-holding, accessible, mother, age 36 year)*

## 4 Discussion

This qualitative study assessed the knowledge and practices of cattle feed processors, retailers, extension workers, dairy farmers, and caregivers of young children about feed availability, storage practices, aflatoxin risks, child dairy consumption, and dairy sales. We found that animal feed processors and their agents had relatively good knowledge about aflatoxin, but farmers and retailers did not. Animal feed storage conditions were generally poor, and testing for aflatoxin was essentially inaccessible or uneconomical. The consumption of milk was limited to skim milk as butter was the main source of household income. Surprisingly, and disconcertingly from a nutrition perspective, we found that the high cost of dairy products also led households – including dairy producers – to dilute skim milk with water before feeding children. Such practices can lead to reduced nutrient supply from milk and increase the risk of microbial contamination that can in turn increase the risk of malnutrition. Malnourished children with weakened immune system also have a high susceptibility to the harmful effects of aflatoxin.

The limited knowledge about aflatoxin and the inappropriate feed storage conditions observed in this study are quite concerning as they affect the health and productivity of the cows and pose risks to the health of consumers [28,29]. Although farmers understood that feeding moldy feed was not good for the cows, the linkage to human health was not clear to them. Consequently, the mitigation strategies adopted were to sun-dry moldy feed ingredients, wash affected parts, and mix spoiled grains with good ones. Some of these practices can further exacerbate the contamination of the feed as they increase the moisture content [30]. Indeed, several studies conducted in Ethiopia showed high levels of aflatoxin M1 in milk [22,24], also translating to high aflatoxin M1 concentrations in human breastmilk [28]. AFM1 contamination has also been linked to feed storage conditions and feed type [23].

Owing to significant supply constraints, animal feed prices in Ethiopia have skyrocketed since 2020 [31]. As a result, small-holding dairy farmers have limited scope to afford commercial animal feed and relied on grass, corn stalks, and other locally available feed options. As a result, farmers may prioritize feed quantity and overall affordability and profitability over feed quality. Consequently, the degree of aflatoxin contamination is likely to show seasonal variation and may vary by herd size. More, not all feeds are equally susceptible to aflatoxin contamination. For example, an earlier study showed higher contamination when *noug* cake was used as feed [23].

These findings will have implications for the monitoring of feed and milk quality, the technologies to be chosen or adopted to reduce aflatoxin, as well as the type of education to be provided to actors along the value chain. For example, regulatory agencies may need to adapt their sampling strategies to take samples in different seasons. To be effective, efforts to improve the quality and safety of feed and milk through education or adoption of technologies would also benefit if they followed a targeted approach based on herd size and market accessibility (e.g., rural vs. peri-urban). Also, human aflatoxin exposure may vary by season and geographic location, and thus assessment of exposure should also account for these variations to design more effective public health strategies [28].

In line with earlier quantitative studies conducted in Ethiopia, our qualitative study indicated that milk consumption among children was relatively low or infrequent despite widespread cattle ownership [32]. However, this study revealed that the true nutrient intake from milk may be even lower than expected because, in this study setting at least, milk was given to children after extracting butter, and often are diluting the skim milk with water. Our observations of the traditional process of extracting butter revealed unhygienic practices in milk handling such as no handwashing and unclean storage material used during and after extraction of butter, likely increasing the risk of microbial contamination and risks of food-borne diseases such as diarrhea. Diluting milk with water can also further increase the risk of contamination, particularly in settings such as rural Ethiopia where water quality is a major issue [33]. The fact that households dilute their own milk supply with water was surprising because most studies only document contamination of dairy products within commercial value chains [21]. Here, evidence of contamination of dairy products by the household itself suggests that policymakers should consider the food safety risks associated with milk production and consumption within the household, not just commercial value chains, such as through behavioral change communications interventions or agricultural extension efforts.

The present study has a number of strengths and limitations that need to be considered when interpreting the findings. First, this study provides a detailed assessment of the safety risks associated with the dairy value chain in selected sites in southern Ethiopia. The study included interviews and focus group discussions with health extension workers, agriculture experts, feed processors, feed traders, dairy farmers, and mothers/caregivers of children under two years of age. However, feed availability and storage practices are likely to vary by location, suggesting that the present findings may not reflect practices in other parts of the country, although there likely are many similarities based on common constraints like low incomes, the high cost of feed, seasonality, et cetera. Another limitation is that only two feed processors operating in the study area were identified and interviewed. Although we were able to assess their knowledge, the findings may not be extrapolated to other feed processors in the country.

Notwithstanding the above limitations, this study provides insight into the knowledge and practices surrounding aflatoxin contamination in the dairy value chain in Ethiopia. The study highlighted the knowledge gap regarding aflatoxin and proper feed storage. Moreover, the study indicates that factors such as cost, seasonality, accessibility, and herd size influence the choice of animal feed and hence the potential risk of aflatoxin exposure. Lastly, the dilution of milk with water even for consumption within dairy-producing households and the possibility of food safety risks associated with such dilution is, to our knowledge, novel information. Such information can help inform future exposure assessments take these practices.

Overall, these findings underscore the need for increased awareness and improved practices to prevent aflatoxin contamination in animal feed and promote safe milk consumption. Addressing the gaps in knowledge, improving storage conditions, and ensuring the availability and 3affordability of quality feed is essential for safeguarding the health and well-being of both animals and consumers in the dairy value chain in Ethiopia. To increase the consumption and safety of milk, efforts that increase access to affordable and quality animal feeds are needed. Improving storage conditions, the promotion of hygienic processing and milk handling, and regular testing for aflatoxin and water quality used in milk dilution should complement and inform ongoing nutrition education activities.

## CRediT authorship contribution statement

**DH, KH, FW, MT, and KB:** Conceptualization, Methodology, Writing; **AA, KB:** Investigation, field survey, and formal analysis, as well as writing the first draft; **DH, KH, AP, FW, MT, and KB:** review & editing; All authors have read and approved the final version of the manuscript.

## Declaration of competing interest

None.

## Data Availability

This is not relevant as this is a qualitative study
